# Short review on human umbilical cord lining epithelial cells and their potential clinical applications

**DOI:** 10.1186/s13287-017-0679-y

**Published:** 2017-10-10

**Authors:** Razwa Saleh, Hasan Mahmud Reza

**Affiliations:** Department of Pharmaceutical Sciences, North South University, Plot 15, Block B, Bashundhara, Dhaka, 1229 Bangladesh

**Keywords:** Cord lining cells, Mesenchymal stem cell, Epithelial stem cell, Wound healing, Ocular therapy

## Abstract

**Background:**

The human umbilical cord has been studied extensively in the past two decades. It is free of ethical dilemmas, non-tumorigenic, and less immunogenic and thus provides a significant advantage over other stem cell sources. The cord lining yields both mesenchymal and epithelial stem cells. The mesenchymal cells have been appraised at length by many researchers, which led to the current review focusing on the cord lining epithelial cells (CLECs). These cells have high proliferative capacity and their superior harvest and multiplication, using the revolutionary CellOptima^TM^ technology, makes them better candidates in comparison to contemporary adult stem cells. Following 30 replication cycles these cells have been observed to retain their stemness, with their phenotype and karyotype intact. However, their remarkable immunosuppressant properties, protecting self as well as co-transplanted allografts from rejection, are what truly define their transplantation potential. They have been successfully applied to many chronic conditions, using animal models, including type 1 diabetes, limbal stem cell deficiency, burn injuries, and wound healing, etc. with encouraging results.

**Conclusions:**

This review first discusses some of the advantages afforded by CLECs over other stem cell lines and then delineates their potential use in various clinical applications. Clinical trials using CLECs are currently underway in the US in collaboration with CellResearch Corp. and their potential positive findings will help garner an FDA approval, likely leading to the eventual commercialization of this promising technology.

## Background

Regenerative medicine focuses on the use of stem cells for the repair and replacement of damaged tissues and organs with the future prospect of curing chronic disorders. This involves the use of stem cells from different sources to generate specific cellular lineages through directed differentiation which can be applied to damaged environments for a speedy recovery [1]. Regeneration aims not only to restore fully functional tissues but also to counter congenital abnormalities where, to begin with, normal physiological functioning was absent [2].

Embryonic stem cells were first considered as a viable source for research [3]. However, several obstacles, including the ethical objections and possible generation of teratomas or imprinting disabilities, have prompted researchers to explore alternative sources of stem cells [4]. One practicable source to be considered is the umbilical cord lining. Stem cells from the umbilical cord lining are multipoint, possess high proliferative capacity, and have also been discovered to be immunologically naïve, making them ideal candidates for regenerative therapy [5].

Hematopoietic, epithelial, and mesenchymal cells have been isolated from umbilical cord blood, while both epithelial and mesenchymal cells have been collected from the Wharton’s jelly [6], amniotic fluid [7], amniotic membrane [8], and cord lining [5] using various techniques. Although the umbilical cord lining cells have been known to express both mesenchymal and epithelial stem cell markers, they have also demonstrated positive expression of certain embryonic stem cell markers, placing their differentiation capabilities somewhere in between embryonic and adult stem cells [9].

This review is directed at describing the various advantages the umbilical cord lining-derived epithelial cells have over other promising stem cell types and also explores their potential clinical applications.

## Cord lining epithelial stem cells

**Fig. 1:**
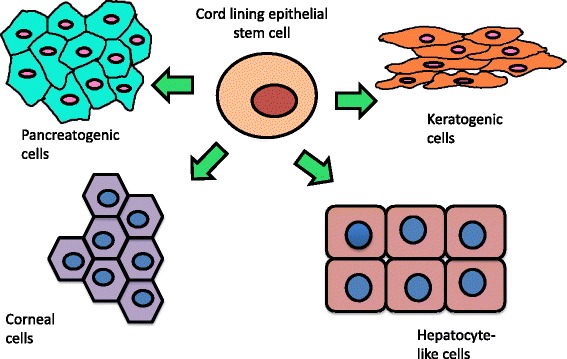
Differentiation potential for cord lining epithelial cells

The richest source for umbilical cord-derived stem cells is the cord lining. Umbilical cords are collected from healthy women undergoing surgery and who have not been diagnosed with any infectious diseases like hepatitis, HIV, etc. The tissue is then cut into 2-cm segments, washed, disinfected with antibiotic mixture, and further cut into small squares of 0.5 cm for cell isolation by explant culture using specific media. The cord contains both mesenchymal and epithelial cells [5, 9]. The epithelial cells are currently under investigation for their use in a wide range of applications from wound healing to ocular surface regeneration and much research is currently underway to explore the full potential of this multipotent cell population. Another unique cell type, coined mucin-expressing cord lining epithelial cells (CLECs-muc), has been isolated by Reza et al. [9]. These display higher colony-forming efficiency, proliferative potential, and passaging ability and express both embryonic and adult stem cell-specific genes. Similar to embryonic stem cells, they express OCT-4, NANOG, SSEA-4, REX1, and SOX2, to which their stem cell like properties can be attributed. While predominantly expressing the epithelial MUCIN1 and cytokeratins, they also express the mesenchymal stem cell surface marker CD166. Furthermore, they possess distinctive p63 expression profiles. Treatment of CLEC-muc cells with BMP4 results in their differentiation into precursor non-keratinized epithelial cell types via regulation of nuclear p63 gene expression. This novel cell type is likely to play a critical role in future clinical trials, namely in ocular surface regeneration therapy [10]. Immunological characterization further detected both the classic human leukocyte antigen molecules HLA-A, B, and C and the non-classic MHC class I molecules HLA-G and HLA-E in CLECs. These non-classic molecules are key contributors to immunosupression via modulation of T cells, dendritic cells, and natural killer cells, observed through responses to mixed leukocyte reaction (MLR) assays and the use of anti-HLA-G and E antibodies [14]. This immunoregulatory response also contributes to CLECs’ ability to prolong the survival of co-transplanted cells such as keratinocytes [14]. The differentiation capability of CLECs is shown in Fig. 1.

Certain embryonic and induced pluripotent stem cell properties have impeded their widespread clinical acceptance. The properties of umbilical cord lining cells are better in some regards compared to these pluripotent cell types (elaborated in Table 1); a comparison with other extra-embryonic tissue is provided in Table 2.

**Table 1:** Comparison of umbilical cord lining epithelial cells with other contemporary stem cell sources

	ESCs	Induced pluripotent stem cells (iPSCs)	Bone marrow-derived stem cells (adult stem cells)	Umbilical cord lining epithelial stem cells
Source	• Pluripotent stem cell source • Controversial sourcing • Requires invasive procedure	• Pluripotent cells • Reprogrammed adult blood or dermal fibroblast cells • Inefficient generation procedure (0.01–1%) • Relatively expensive autologous transplantation procedure	• Multipotent stem cells • Derived using an invasive procedure • Potential risks and significant discomfort to the donor • Limited proliferative capacity • Aging at each doubling	• Multipotent stem cells • Source is medical waste and inexpensive • Devoid of maternal or fetal morbidity • Billions of cells can be isolated from the primary cell source. • Stemness retained after 30 replication cycles with their phenotype and karyotype intact
Tumorigenicity	• Characteristically produce teratomas • Aneuploidy and accumulation of oncogenic mutations over multiple replication cycles produce aggressive teratocarcinomas	• MYC used in cell line generation is oncogenic • Cell line generation through viral vectors can activate integration site-adjacent oncogenes or suppress the tumor suppressor genes • Possible precursor adult cell epigenetic memory retention • Teratomas from undifferentiated cell contamination in the final product	• Genetically modified cells can generate teratomas through proto-oncogenic activation	• Highly proliferative but do not produce tumors • Acquiescent to transgene integration sans tumorigenesis
Immunogenecity	In undifferentiated state: • Low immunogenicity • No expression of immunomodulatory molecules such as CD95 and IL-10 In differentiated state : • Immunogenicity is similar to adult fibroblasts • Mouse ESCs acquiescent to syngenic but not allogenic transplantations	Initially, T-cell-mediated autoimmune rejection observed in: • iPSC-derived myocardial, endothelial cells • Teratoma formation models Recently, successful allogenic transplantation of iPSC derived retinal pigment epithelial cells in immune-matched monkeys observed • Clinical study to observe iPSCs’ ability to arrest macular degeneration approved in Japan	• Long history of safety in clinical trials • Possible immunomodulatory properties Possible applications in: • Hematological malignancies and severe aplastic anemia • Autoimmune disorders	• Express MHC class I molecules HLA- A, B, and C as well as the non-classic HLA-G and -E • HLA-G and -E modulate maternal immune response, suppressing T cells, NK cells and dendritic cells • No expression of the co-stimulatory cell surface markers • Ideal for allogenic transplantation • Prolong co-transplanted cell survival

*Abbreviations*: *ESC* embryonic stem cell, *HLA* human leukocyte antigen, *iPSC* induced pluripotent stem cell, *MHC* major histocompatibility complex, *NK* natural killer

**Table 2:** Comparison of umbilical cord lining cells against other extra-embryonic tissue derived stem cells

Source	Cell types	Yield	GVHD	Ease of isolation	Immunomodulation by HLA–G and E	Proliferation and expansion
Umbilical cord blood	HSCs, MSCs, EpSCs,	Low yield of MSCs and ESCs, high yield of HSCs	Lower incidence of chronic GVHD	Low MSC level requires highly refined isolation techniques	No expression	Slow expansion and engraftment
Wharton’s Jelly	MSCs	High	Improvement with acute steroid-resistant GVHD	Possible heterogeneous cell contamination	No expression	Easily and rapidly expanded
Cord lining	CLECs, CLMSCs	Highest yield (potentially six billion MSCs and six billion EpSCs per lining)	Dramatic improvements with MSCs without additional immunosuppressant use	Time-consuming isolation process	CLECs express HLA-G and E CLMSCs express insignificant amounts	Highest proliferation and migration capacity
Placenta	AMSCs, AECs, DSCs	Low	Placental DSCs relatively effective against acute and chronic GVHD	Longer isolation process compared to cord-derived cells	Expresses HLA-G and E	Limited proliferation capacity of hAECs

*Abbreviations*: *AMSC* amniotic mesenchymal stem cell, *AEC* amniotic epithelial stem cell, *CLEC* cord lining epithelial stem cell, *CLMSC* cord lining mesenchymal stem cell, *DSC* decidual stromal cell, *EpSC* epithelial stem cell, *GVHD* graft versus host disease, *HSC* hematopoetic stem cell, *HLA* human leukocyte antigen, *MSC* mesenchymal stem cell

### Potential clinical applications of CLECs

**Fig. 2:**
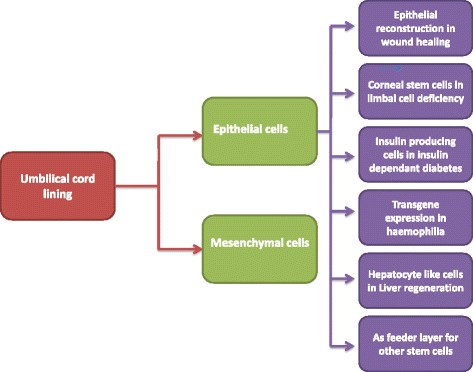
The potential clinical applications of epithelial cells derived from umbilical cord lining

Many successful investigations have been carried out on animal models using CLECs, with encouraging results. All published studies concerning CLECs are listed in Table 3. Figure 2 demonstrates the currently researched fields in which cord lining cells have been examined for applicability.

**Table 3:** Studies outlining the progress and understanding of umbilical cord lining-derived epithelial cells published since the year 2000

Year	Author	Primary findings
2006	Lund et al. [46]	Human umbilical cord tissue in photoreceptor rescue
2008	Reutze et al. [18]	Comparison between CLECs and epidermal keratinocytes
2009	Sivalingam et al. [30]	Transgene integration in CLECs
2009	Branski et al. [16]	Stem cell therapy in cutaneous wound healing
2011	Zhou et al. [14]	Characterization and transplantation potential of CLECs
2011	Reza et al. [9]	Characterization of a novel cell line, CLEC-muc
2011	Huang et al. [12]	CLECs potential for epidermal reconstitution
2011	Reza et al. [10]	CLEC-muc in ocular regeneration
2012	Huang et al. [11]	Updated review on stem cell applications in burns and wounds
2012	Liras et al. [27]	Advanced therapies for hemophilia including transgene integration in CLECs
2013	Cheong et al. [38]	Derivation of hepatocyte-like cells from CLECs
2014	Lim et al. [5]	Review on CLSCs
2014	Cai et al. [47]	Characterization of the immunological properties of CLECs for allotransplantation
2014	Zhou et al. [40]	Hepatic-like cloned CLECs show potential in regeneration of hepatectomized liver
2015	Ang et al. [45]	CLEC-muc as novel feeder layer for human stem cells
2016	Sivalingam et al. [31]	Successful zinc finger nuclease-mediated integration and secretion of FVIII in CLECs for treatment of hemophilia A

The studies relate to the characterization of the cord lining epithelial cell types, their various uses investigated in the past decade in terms of contribution to possible cure of chronic disorders, possible applicability in conjugation with gene therapy, and lastly their use as feeder layers for supporting other stem cell lines. *Abbreviations*: *CLEC* cord lining epithelial cell, *CLSC* cord lining stem cell, *CLEC*-*muc* Mucin1-expressing cord lining epithelial cells

### CLECs in burn injury and wound healing

In burn injuries, stem cells are used with the aim of achieving improved quality of healing, as the therapies are relatively labor-intensive, time-consuming, and expensive. The objectives include more rapid healing with the potential for regeneration of completely functional tissue, avoiding scar tissue formation or infections by managing the inflammatory response following injury, and ameliorating patient response to regenerative therapy [11]. Multiple stem cell sources have been explored, with skin substitutes developed from embryonic stem cells and induced pluripotent stem cells being impracticable in terms of cost effectiveness [11]. Both epithelial and mesenchymal cells have been examined for their wound healing capabilities, with CLECs proving to be a viable source for epithelial reconstruction [12].

Use of umbilical cord lining tissue has certain advantages over keratinocytes. CLECs can be expanded rapidly and passaged in culture [11], while in vitro keratinocyte expansion is relatively slow. CLECs express MHC class I but no MHC class II proteins, no co-stimulatory factors (CD-40, CD-80, CD-86) and low levels of the IL-1 family and TNF-β1, which reduces inflammatory and fibrotic responses [13, 14]. Higher expression levels of HGF and IL-4 in CLECs promote angiogenesis and keratinocyte proliferation and migration, enhancing wound healing [13]. Additionally, CLECs are immune-privileged cells which participate in acceleration of wound healing through promotion of growth factor VEGF [15] induced neovascularization and paracrine effects [11, 16].

CLECs express cytokeratin patterns similar to keratinocytes (including CK8, CK14, CK16, and CK19) and they were successfully utilized in reconstructing an organotypic skin equivalent, including the stratified epithelium [12]. They have been proven to be a viable substitute for keratinocytes and can also serve as an adjunct acting as an epidermis-like layer protecting the wound site prior to treatment with the permanent graft [17, 18].

### CLECs in limbal stem cell deficiency

The limbus, a region lying between cornea and conjunctive, is considered as the reservoir of ocular stem cells. The preservation of ocular functionality and integrity is by virtue of the limbal stem cells located in the basal region of the limbus [19]. A handful of conditions, including Steven Johnson’s syndrome and injury associated with chemical burns, have culminated in limbal stem cell deficiency (LSCD) resulting in severe visual impairment [20]. The conventional treatment involves cornea transplantation, which has generally produced a poor prognosis [10]. An alternative treatment strategy involves autologous or allogenic transplantation of adult limbal stem cells directly to the damaged limbus, which has generated encouraging results [21, 22]. However, obtaining the required quantities of adult stem cells necessary for therapy has proven to be a challenge. This led to the use of conjunctival stem cells [23] and oral mucosal cells [24] as a scaffold for regenerating ocular surface damage with varying degrees of success.

Both mesenchymal and epithelial cells from cord lining have been investigated for their corneal regenerative capacity, with epithelial cells showing promising results. A study conducted by Reza et al. [10] tested the effectiveness of using a novel cell line derived from the cord lining in treating limbal stem deficiency as an alternative to limbal stem cells. A previous study demonstrated that CLEC-muc express certain putative markers attributed to limbal stem cells, including p63, ABCG2, BMI1, and HES1 [9]. The CLECs-muc sheet was transferred on a human amniotic membrane (HAM) scaffold which was then grafted onto the eyes of limbal stem cell-deficient rabbits. The results were very encouraging, showing minimal peripheral neovascularization and opacification. Not only was no immunorejection observed, but the cells were also non-tumorigenic, easily expanded on HAM, and eventually differentiated into cell populations which were phenotypically similar to corneal stem cells. The cells also produced the corneal markers CK3/12 over the conjunctival markers CK4 and CK19, which was a major improvement in contrast to the eyes transplanted with plain HAM, which were re-epithelialized by conjunctival epithelial cells.

### CLEC differentiation to insulin-producing cells in type 1 diabetes mellitus

Type 1 diabetes is a debilitating disorder which requires life-long exogenous insulin administration. Whole pancreatic allograft from cadaveric donors has succeeded in a number of clinical trials to restore the normal physiologic control of hyperglycemia, but this treatment has major shortcomings [25, 26]. Firstly, the surgery itself has a high morbidity rate, and secondly, even following successful surgery, the patient has to remain on life-long immunosuppressant therapy, which is inherently associated with side effects, severely downgrading the recipient’s quality of life. As the most versatile cell, both mouse and human embryonic stem cells have received much attention and have been successfully differentiated into insulin-producing cells. However, the ethical dilemmas and immunological issues once again arose. Studies on umbilical cord blood proved to be unfeasible due to the limited number of mesenchymal or epithelial cells derived from it. However, the cord lining revealed an abundance of both cell types, making it an ideal trial candidate. The undifferentiated CLECs are believed to reside in the embryonic stage whereas the CLMCs are believed to be further along the developmental tree, at the posterior foregut stage. A study conducted by Zhou et al. [14] explored not only the immunological advantage afforded by the CLECs, but also the therapeutic benefits of modified CLECs on streptozotocin-induced diabetic mice over 3 weeks. A lentiviral vector containing an eGFP fluorescent transgene and human proinsulin gene, connected by a ribosome entry site linker, were transduced into CLECs. Even after ten rounds of passaging the successfully transfected cells continued to produce eGFP and human C peptide (indicator of insulin production). These cells were resuspended in RPMI-1640 medium and a single dose of 250 μl of cell solution (1 × 10^7^ cells per mouse) was then intraperitoneally injected into a streptozotocin-treated diabetic mouse model. Both treatment and control groups had their blood glucose level tested once a day in the first week and twice a day for the next two weeks following which the mice were euthanized. An opaque cluster of transgene-expressing CLECs were found, proximal to the damaged pancreas, in the cell-treated group of mice. In this pilot study, the test subjects showed a statistically significant alteration in the hyperglycemic condition over 18 days post-stem cell therapy compared to the controls. These cells thus display therapeutic potential for treating diabetes mellitus proven through both in vitro and in vivo studies [14].

### CLECs with transgene integration in the treatment of blood disorders such as hemophilia

The majority of hemophilia cases are attributed to mutations in clotting factor VIII or factor IX [27]. A key problem with the available factor replacement therapy is the development of antibodies against the proteins, rendering these factor substitutes useless [28]. Also, factor replacement therapies prove to be too expensive for most patients [27].

Since hemophilia is a monogenic disorder, it is amenable to factor replacement as well as gene therapy. Several studies have reported immune responses with the currently most effective adenoviral vector-related therapy. After consideration of adult stem cells, autologous fibroblasts, platelets, and hematopoietic stem cells, the umbilical cord lining epithelial cells have also been recently considered.

Kermani et al. [29] discussed the stable transduction and expression of trangenes by cord lining cells. After transgene incorporation into cells, their transcriptional profiles were examined to determine transcriptome level alterations. A 1-Mb window centered on the integration sites were monitored for altered gene expression. Around 15 oncogenes and tumor suppressor genes were located around the integration sites and none showed any significant increase or decrease in expression. The modifications of the CLECs had no influence on the genome copy number and there was also no evidence to suggest that genetic integration favored chromosomal rearrangements. These assays confirmed the stability of the transgene-incorporated CLECs. The transgene was successfully expressed in vitro, and its in vivo expression was also observed in a hemophilic mouse model. This study has paved the way for further research into the use of CLECs, which provides a non-genotoxic cell-based therapy enabling allogenic application without the danger of immune rejection or teratoma formation [30].

A recent study also investigated AAVS1 locus-specific, programmable, non-viral zinc finger nuclease (ZFN)-directed FVIII transgene integration in primary human CLECs [31]. Three different constructs of ZFN AAVS1 were tested and the Enhanced Sharkey version, exposed to transient hypothermia (30–37 °C), generated the best possible results in terms of FVIII transgene integration, as well as stable secretion of FVIII by the primary CLECs. Using whole genome sequencing, no functionally significant chromosomal rearrangements were observed. RNAseq also revealed that no potential oncogenic alterations were produced using this technique. Bone marrow-derived stromal cells alongside CLECs were also tested using this technique, with both showing comparable FVIII expression. However, with the significant advantages CLECs possess over adult stem cells in terms of source, scale-up, and immunosuppressive properties, the former could prove to be a strong contender for transplantation therapy. This ZFN technology has already shown promising results in phase 1 clinical trials using T cells [32] and will likely prove beneficial, coupled with CLECs, for pediatric and adult patients alike.

### CLECs differentiated into hepatocyte-like cells in end-stage liver diseases

Hepatic failure due to various disease conditions resulting in end-stage liver disease often requires hepatic transplantation. Grafting of hepatocytes rather than whole liver transplantation has proven to be a more effective treatment strategy. Since terminally differentiated hepatocytes are almost impossible to maintain in culture, stem cells are of critical importance in terms of clinical applications.

Non-hepatic origin stem cells considered for grafting include bone marrow cells [33], umbilical cord blood cells [34, 35], as well as embryonic cells [36]. Embryonic stem cells naturally were the primary candidates for hepatic transplantation, but understandably the ethical issues surrounding their use obligated scientists to look for alternatives which preferably did not involve donor morbidity. Umbilical cord-derived stem cells proved to be a worthy alternative which did not require any genetic manipulation.

Cheong et al. [37] demonstrated that CLECs in an undifferentiated state express certain hepatic cell gene markers such as albumin, alpha fetoprotein (AFP), and cytokeratins 18 and 19 [37]. In order to facilitate hepatocyte differentiation, these freshly isolated CLECs were cultured in a hepatocyte culture medium supplemented with essential reagents and their transformation was observed. The CLECs underwent both structural and functional changes during the culture period, analysis being performed every 7 days for 28 days. This study observed a morphological change in the CLECs, which started off as cuboidal epithelial cells that eventually morphed into a rounded version, at which point the cells were transferred into a hepatic maintenance medium (HMM), culminating in the final transformation of all culture cells into a circular or oval shape resembling primary hepatocytes. Functional modifications involved further gene expression of tyrosine amino transferase [38], hepatocyte nuclear factor 4 alpha (HNF4A), hepatocyte nuclear factor 1 beta (HNF1B), factor VIII and CYP3A4. These hepatocyte-like differentiated CLECs secreted albumin, produced urea, stored glycogen, and performed uptake of low-density lipoproteins, albeit with temporal variations in activity. The CYP3A4 expression would even allow drug metabolism [37].

Takashima et al. [39] reported that human amniotic mesenchymal stem cells may influence albumin production by human amniotic epithelial cells, and Cheong at al. [37] successfully isolated the cord lining epithelial cells which continued to produce albumin on differentiation. Although certain issues were encountered involving decrease in functionality on transferring the cells to HMM, the umbilical cord lining-derived epithelial cells deserve practical consideration for use in clinical applications.

In a further assessment of CLECs, Zhou et al. evaluated the hepatoregenerative capacity of hepatic-like cloned cord lining epithelial cells in a porcine model. The treatment group received a tissue fleece lined with hepatic-like cloned CLECs and daily injections of CLECs for 7 days. The CLEC-treated livers showed up to 89% recovery of the original liver volume while the control subjects showed 67–75% liver regeneration, demonstrating their significant role in hepatic regeneration [40].

### CLEC-muc used as feeder cells

In order to maintain cell pluripotency or stemness, certain feeder layers or conditioned medium have to be used for the culture of stem cells. The conventional use of mouse 3 T3 cells or mouse embryonic fibroblasts as feeder layers pose the risk of zoonotic infection [41]. Various human cell lines have been used as feeder layers, including bone marrow-derived stromal cells, breast skin fibroblasts [42, 43], and foreskin cells from circumcision of newborns [44]. These have been compared with a mouse feeder layer co-culture system. The CLECs have similarly been assessed for their possible application as a feeder layer in the growth of limbal epithelial stem cells and their efficiency was compared against the mouse 3 T3 feeder cells. The cells generated growth of limbal stem cells, which produced the putative markers HES1, ABCG2, deltaNp63, and BMI1 with the same efficacy as the mouse feeder layer. They also produced the cytokeratins CK14, CK15, CK19, and CK3, which are typical of corneal epithelial stem cells. This success extends an opportunity for further analysis with other stem cell types and CLECs as a feeder layer, protecting against zoonotic infections while sustaining robust growth of cells [45].

## Conclusions

This review briefly introduces the currently available therapies for various chronic conditions followed by their treatment limitations and possible avenues for subsequent cell-based therapies which can be explored for their potential clinical applications. It delineates the various prospective stem cell sources examined thus far and makes a comparison with the CLEC-centered approach. This includes providing evidence-based implications for the future of CLECs in the treatment of ocular disorders, insulin-dependent diabetes, liver failure, wound regeneration, and various other conditions for which other stem cell sources have presented different limitations in their applicability. These cells are ethically acceptable, easily accessible, immunologically naive, and non-tumorigenic and a considerable amount of resources have been directed in many countries for the long-term storage of their source material.

## Abbreviations

ABCG2: ATP-binding cassette sub-family G
BMI1: B lymphoma Mo-MLV insertion region 1 homolog
BMP4: Bone morphogenetic protein 4
CD: Cluster of differentiation
CK: Cytokine
CLEC: Cord lining epithelial stem cell
CLEC-muc: Mucin-expressing cord lining epithelial cell
CLMC: Cord lining mesenchymal stem cell
CT: Computed tomography
CYP3A4: Cytochrome P450 3A4
eGFP: Enhanced green fluorescent protein
HAE: Human amniotic epithelial cells
HAM: Human amniotic membrane
HES1: Hairy and enhancer of split-1
hESC: Human embryonic stem cell
HGF: Hepatocyte growth factor
HLA: Human leukocyte antigen
HMM: Hepatic maintenance medium
HNF1B: Hepatocyte nuclear factor 1 homebox B
HNF4A: Hepatocyte nuclear factor 4 alpha
IL: Interleukin
KLF4: Krupple like factor 4
MESC: Mouse embryonic stem cell
MHC: Major histocompatibility complex
MYC: Mitomycin c
OCT-4: Octamer binding transcription factor 4
REX-1: Reduced expression 1
SOX2: Sex determining region Y box 2
SSEA-4: Stage-specific embryonic antigen
TNF-β1: Tumor necrosis factor beta-1
VEGF: Vascular endothelial growth factor
ZFN: Zinc finger nuclease
